# Taxonomic revision of the *Ligularia fischeri* complex (Asteraceae) in Korea

**DOI:** 10.3897/phytokeys.281.211143

**Published:** 2026-11-02

**Authors:** Minsu Park, Nuree Na, Chang-gee Jang

**Affiliations:** 1 International Biological Material Research Center, Korea Research Institute of Bioscience and Biotechnology, Daejeon, Republic of Korea International Biological Material Research Center, Korea Research Institute of Bioscience and Biotechnology Daejeon Republic of Korea; 2 DMZ Forest Biological Resources Research Division, Korea National Arboretum, Yanggu, Republic of Korea DMZ Forest Biological Resources Research Division, Korea National Arboretum Yanggu Republic of Korea; 3 Department of Biology Education, Kongju National University, Kongju, Republic of Korea Department of Biology Education, Kongju National University Gongju Republic of Korea

**Keywords:** Indumentum, mixed gathering, nomenclatural priority, second-step lecto­typification, Tussilagininae, vernacular names

## Abstract

A taxonomic revision of the *Ligularia fischeri* complex (Asteraceae, Senecioneae) in Korea was conducted to clarify the identity and systematic position of problematic taxa, particularly those commonly recognized as ‘Gom-chwi’. Based on extensive herbarium reviews and micromorphological analyses of stem trichomes using scanning electron microscopy (SEM), the complex was confirmed to consist of morphologically and ecologically distinct entities. *Ligularia splendens* is distinguished from *L. fischeri* by its later flowering period, uniformly ovate leaflike bracts throughout the synflorescence, and persistent fine arachnoid hairs on the stems. A nomenclatural review revealed that *L. euodon* Miq. (1867) has priority over *Senecio splendens* H.Lév. & Vaniot, (1910), the basionym of *L. splendens* (H.Lév. & Vaniot) Nakai; therefore, *L. euodon* is recognized as the correct name for this taxon. Furthermore, the original material of *L. fischeri* var. *spiciformis* Nakai was found to be a mixed collection comprising both *L. fischeri* s. str. and *L. euodon*. To maintain nomenclatural stability, a lectotype is designated for *L. fischeri* var. *spiciformis* (Nakai 15777, TI00011066 & TI00011067), effectively placing it in synonymy under *L. fischeri*. Updated synonymies and taxonomic notes are provided, along with proposed Korean names for the recognized taxa.

## Introduction

Scientific and vernacular plant names operate under different conventions. The application and correct choice of a scientific name are determined by nomenclatural types, priority, and taxonomic circumscription, whereas vernacular names arise from regional usage and can remain associated with a misidentified or broadly circumscribed taxon. Consequently, taxonomic revisions can disrupt the link between a familiar Korean name and the biological entity to which it is applied. Several Korean cases illustrate this problem. The name “Mindeulle” was long associated with *Taraxacum platycarpum* Dahlst., now considered endemic to Japan, although Korean plants bearing this name correspond to *T. mongolicum* Hand.-Mazz. (Park 2021). In *Broussonetia*, taxonomic clarification led to the application of “Dak-namu,” the plant used for traditional Hanji paper, to *B. × hanjiana* M.Kim and the renaming of *B. × kazinoki* Siebold as “Aegi-dak-namu” (Yun and Kim 2009). Conversely, the rediscovery of natural populations of *Chrysosplenium grayanum* Maxim. in Korea after it had been regarded as endemic to Japan demonstrates the risk of reallocating a familiar common name before taxonomic identity and distribution are adequately verified (Choi et al. 2020). These examples underscore the need to stabilize the association between Korean vernacular names and accurately circumscribed taxa.

Within this context, *Ligularia* Cass. (Asteraceae, Senecioneae, Tussilagininae) is an appropriate case study. The genus comprises approximately 140 species of perennial herbs distributed mainly in Asia, with a few species in Europe, and is particularly diverse in China (Liu and Illarionova 2011). Species are characterized by alternate leaves, commonly with large and long-petiolate basal blades, yellow radiate or discoid capitula arranged in racemose, corymbose, or paniculate synflorescences, and cypselae bearing a bristly pappus (Liu and Illarionova 2011). Here, the *L. fischeri* complex is used in an operational sense for East Asian plants historically treated as *L. fischeri* (Ledeb.) Turcz. or closely allied taxa and sharing large cordate basal leaves, racemose synflorescences, 8–10 phyllaries, ligulate florets, and a light-brown pappus. Members assigned to this complex have been reported from the Korean Peninsula, Japan, northeastern China, the Russian Far East, and Mongolia, but their delimitation differs among regional treatments (Koyama 1966, 1968; Liu and Illarionova 2011; Illarionova 2013).

In Korea, plants commonly called “Gom-chwi” have generally been identified as *L. fischeri* and are widely used as a high-value edible wild vegetable. Nevertheless, the taxonomic identity of these plants remains uncertain. *Ligularia splendens* (H.Lév. & Vaniot) Nakai, described from Jeju Island, has been accepted as a distinct species by some authors (Léveillé 1910; Nakai 1952; Pojarkova 1961; Im 1999; Illarionova 2013; Kozhevnikov et al. 2019) but treated as a synonym of *L. fischeri* by others (Kitamura 1942; Koyama 1968; Lee 1996a, 1996b; Chang et al. 2014; Im 2020). Even major regional floras differ in their treatment of these entities (Liu and Illarionova 2011). Koyama (1966) recognized two morphologically distinct ecotypes within this complex: a late-flowering type with arachnoid hairs from moist habitats and an early-flowering type with simple hairs from drier habitats, but did not formally resolve their taxonomic status. Additional nomenclatural problems remain. *Ligularia euodon* Miq., described from Japan in 1867 and later discussed in relation to *L. sibirica* (L.) Cass. and *L. gigantissima* Nakai (Kitamura 1934; Nakai 1944), has subsequently been treated as a synonym of *L. sibirica* without detailed reassessment. *Ligularia fischeri* var. *spiciformis* Nakai, described in 1944 from the Korean Peninsula and still recognized as a Korean endemic in some treatments, also remains poorly understood.

This study integrates field observations, herbarium and type-specimen examination, and macro- and micromorphological evidence to (1) delimit the Korean members of the *L. fischeri* complex, (2) determine the correct name for the taxon previously treated as *L. splendens*, (3) clarify the identity and typification of *L. fischeri* var. *spiciformis*, and (4) propose Korean names that reflect the revised taxonomic circumscriptions.

## Materials and methods

### Plant materials and herbarium review

Plant materials were collected during their flowering and fruiting seasons from 2018 to 2021. Collected specimens were processed as herbarium vouchers, with selected tissues preserved in 70% ethanol for detailed morphological analysis. All voucher specimens collected during the field surveys were deposited in the Herbarium of Kongju National University (KNH).

In addition, extensive herbarium reviews were conducted through personal visits or loans from the following institutions: KH, KB, KRIB, and KWNU in Korea; TUS and MBK in Japan; and LE in Russia. Herbarium acronyms follow Index Herbariorum (Thiers 2026). Type specimens were examined by analyzing high-resolution digital images provided by the institutions or downloaded from their databases. Relevant protologues, taxonomic revisions, and regional floristic treatments of *Ligularia* and the *L. fischeri* complex were consulted to evaluate the taxonomic circumscriptions and nomenclature of the taxa examined (Miquel 1867; Léveillé 1910; Kitamura 1934, 1942; Nakai 1944, 1952; Pojarkova 1961; Koyama 1966, 1968; Im 1999; Liu and Illarionova 2011; Illarionova 2013; Chang et al. 2014; Im 2020). Available label and georeferenced locality data for the specimens included in the distributional analysis are provided in Suppl. material 2. The point-based distribution map was prepared from these georeferenced specimen records.

### Macromorphological observation

Macromorphological characters were observed and measured directly from field-collected and herbarium specimens. Terminology used in the morphological descriptions and comparisons followed Hickey (1973) for leaf characters and Radford et al. (1974) for general vegetative and reproductive characters. Following Liu and Illarionova (2011), the term “leaflike bracts” is used here for the bracts subtending individual capitular peduncles within the racemose synflorescence, whereas “supplementary bracts” refers to the small bracts situated at the base of the capitulum, outside the involucre; both are distinct from the involucral phyllaries. Fine details of the stem trichomes, including their surface morphology and basal cell arrangement, were examined using SEM as described below.

### Micromorphological observation using SEM

To investigate the micromorphology and basal cell arrangement of the multicellular trichomes on the stems of the *Ligularia fischeri* complex, highlighting the morphological significance proposed by Koyama (1966), scanning electron microscopy (SEM) was employed. Segments of the stems were excised from herbarium specimens and mounted onto aluminum stubs using double-sided conductive carbon tape. To enhance surface conductivity and prevent charging, the samples were coated with platinum (Pt) using an ion sputter coater (E-1010, Hitachi) at a current of 15 mA for 100 seconds. The coated specimens were subsequently observed and photographed using a scanning electron microscope (S-3400N, Hitachi) at an accelerating voltage of 15.0 kV.

## Results and discussion

### Morphological study of *Ligularia fischeri* complex

**Figure 1.**
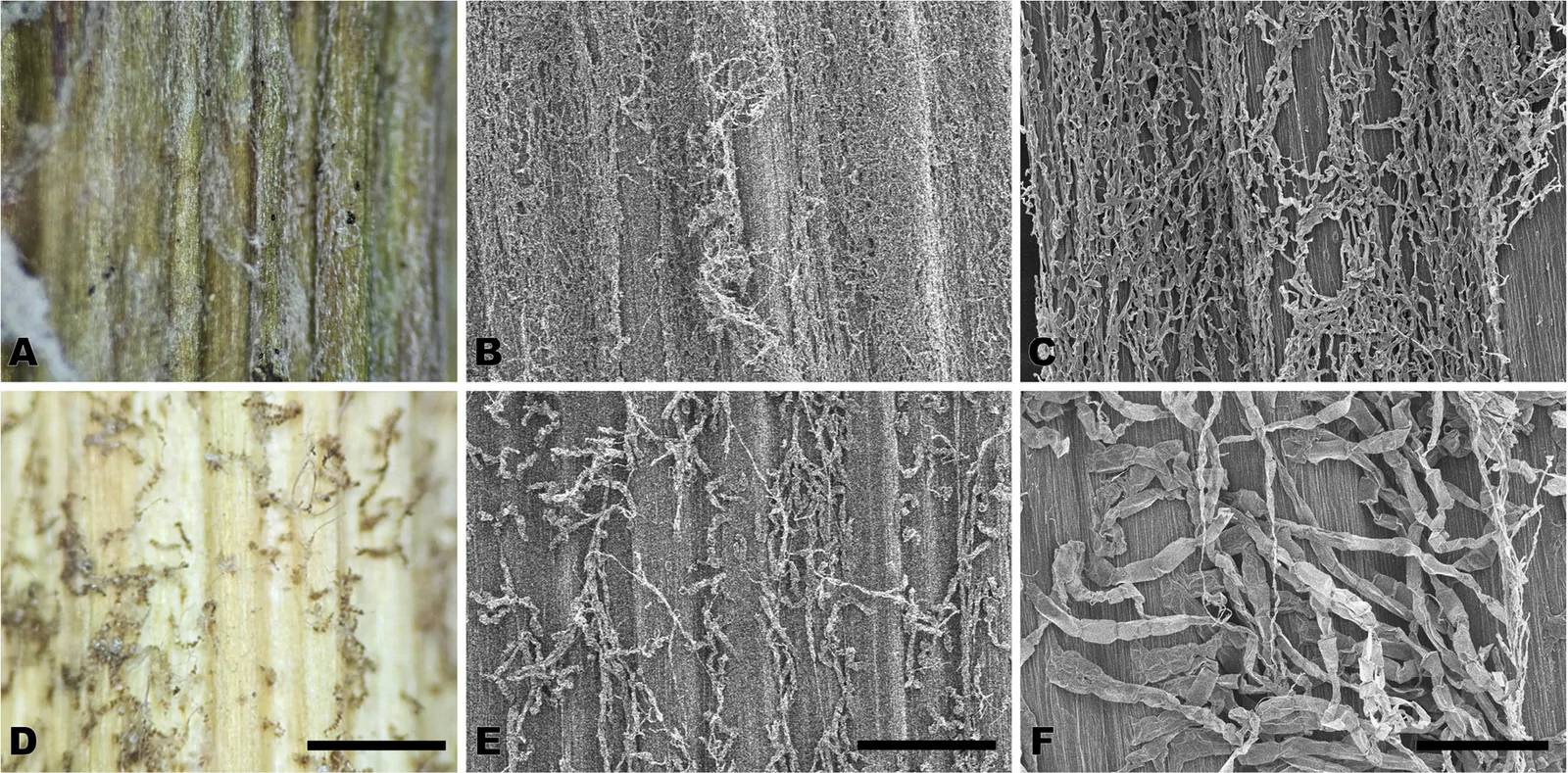
Microscopic images of stems. **A–C**. *Ligularia euodon*; **D–F**. *L. fischeri*. **A, D**. Light micrographs (LM). **B, C, E, F**. Scanning electron micrographs (SEM). Scale bars: 1 mm (**A, B, D, E**); 500 µm (**C, F**).

**Figure 2.**
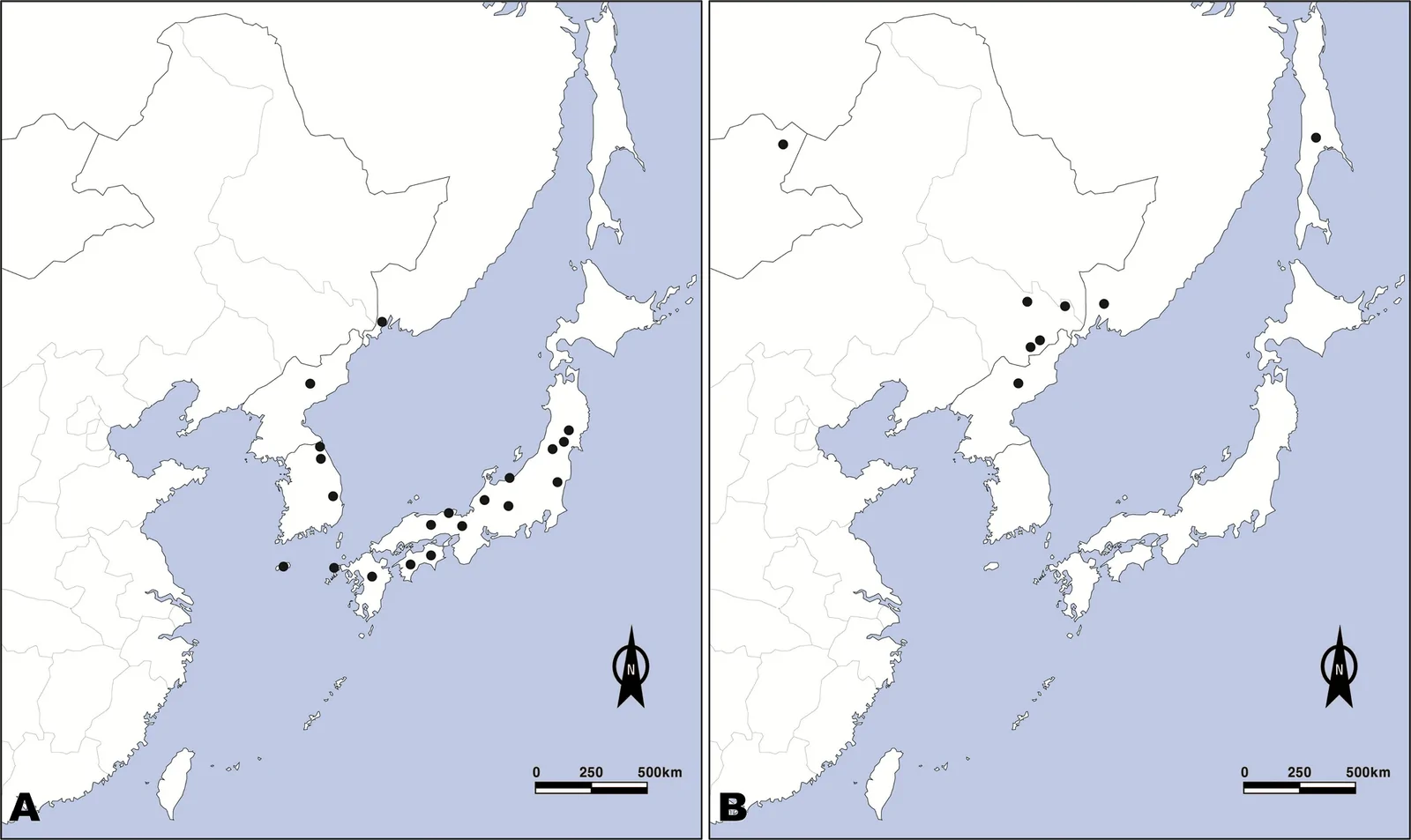
Georeferenced specimen records of *Ligularia euodon* (**A**) and *L. fischeri* (**B**) included in the distributional analysis. Only specimens with sufficiently precise locality information were plotted; therefore, the maps represent the geographic coverage of the available records rather than the complete distributions of the species.

*Ligularia euodon* (previously treated as *L. splendens*) shares morphological similarities with *L. fischeri* in its cordate leaf blades, racemose synflorescence, presence of 8–10 phyllaries and ligulate florets, and light-brown pappus. However, it is clearly distinguished from the latter by several key characters: the flowering period, which occurs later, from August to October (vs. July to August); the bracts of the synflorescence, which are uniformly ovate throughout (vs. shifting from ovate to lanceolate toward the apex); and the lower number of supplementary bracts, typically 2–3 (vs. 2–7). Regarding trichome morphology, the stems and petioles of *L. euodon* are covered with fine arachnoid hairs that persist to varying degrees or eventually become glabrescent (vs. persistently hirsute, with coarse, distinctly septate hairs that are often multiseriate at the base in *L. fischeri*) (Fig. 1). These morphological distinctions align with the geographical distributions of the two species; *L. euodon* is primarily distributed in the Korean Peninsula (including Jeju Island), Japan, and Russia (Primorsky Krai), while *L. fischeri* is found in North Korea, northeastern China, Russia (Primorsky Krai, Sakhalin), and Mongolia. These findings reconfirm the morphological and ecological divergence within the *L. fischeri* complex previously noted by Koyama (1966), specifically validating the distinction between the late-flowering moist-habitat type with arachnoid hairs and the hirsute, early-flowering dry-habitat type. The diagnostic morphological, phenological, and ecological differences between *L. euodon* and *L. fischeri* are summarized in Table 1. The distributions of the two species, based on the georeferenced specimens examined in this study, are shown in Fig. 2.

**Table 1.** Comparison of diagnostic morphological, phenological, and ecological features of *Ligularia euodon* and *L. fischeri*.

**Character**	***Ligularia euodon***	***Ligularia fischeri***
Indumentum of stems and leaf petioles	Glabrous or covered with fine arachnoid hairs, sometimes becoming glabrescent	Densely and persistently hirsute
Trichome morphology	Fine and arachnoid	Coarse and distinctly septate, often multiseriate at the base
Abaxial leaf surface	Sparsely hairy	Densely hairy
Shape of leaflike bracts	Uniformly ovate throughout the synflorescence	Ovate proximally, becoming lanceolate distally
Number of supplementary bracts	2–3	2–7
Flowering period	August–October	July–August
Typical habitat	Moist habitats	Relatively dry habitats

### Taxonomic identity of *Ligularia euodon* Miq.

*Ligularia euodon* was described by Miquel (1867) based on a specimen collected in Japan by Siebold. Although Miquel regarded the specimen as incomplete (“mancum”), he noted several diagnostic characters, including an abaxial leaf surface initially covered with arachnoid hairs but becoming glabrescent (“arachnoideo-adspersa glabrescentia”) and approximately 10 lanceolate phyllaries. The name has subsequently often been placed in the synonymy of *L. sibirica*, but this treatment does not appear to have been based on a critical reassessment of the type. Examination of the holotype at the Naturalis Biodiversity Center (L0002291) confirmed that, despite its partially lost synflorescence, the specimen retains fine arachnoid hairs rather than coarse hirsute hairs, ovate leaflike bracts subtending the remaining capitula, and a light-brown pappus protruding beyond the involucre.

The exclusion of *L. euodon* from the synonymy of *L. sibirica* is further supported by the infrageneric classification of Illarionova (2013). Within sect. *Ligularia*, subsect. *Speciosae*, which includes *L. fischeri* and *L. splendens*, is characterized by a pappus shorter than the corolla of the disc florets, whereas subsect. *Ligularia*, which includes *L. sibirica*, has a pappus as long as or nearly as long as the disc-floret corolla. In the specimens of *L. euodon* examined here, the disc-floret corollas were 11.4–15.8 mm long, and the pappus was 5.8–10.4 mm long, consistently corresponding to the condition of subsect. *Speciosae*. Moreover, the diagnostic characters attributed to *L. splendens* by Illarionova (2013)—broadly ovate leaflike bracts, arachnoid or absent pubescence on the stems, petioles, and phyllaries, and a glabrous or sparsely short-hairy abaxial leaf surface—closely agree with the holotype of *L. euodon* and the material examined in this study. These characters support the exclusion of *L. euodon* from the synonymy of *L. sibirica* and its recognition as conspecific with the taxon previously treated as *L. splendens* within the *L. fischeri* complex.

### *Ligularia fischeri* var. *spiciformis* Nakai

*Ligularia fischeri* var. *spiciformis* was described by Nakai (1944) based on specimens collected from Handaeri, Bujeon Highlands, Hamgyongnam-do, North Korea (15 Aug. 1935, T. Nakai 15777, TI). In the protologue, he briefly noted that this variety resembles the typical form but is characterized by a 20–25 cm-long synflorescence with markedly short peduncles (3–8 mm), resulting in a spike-like appearance. In the specimens examined in this study, capitular peduncles measured from the middle portion of the synflorescence were 10.3–47.9 mm long. Peduncle length varied according to position within the synflorescence, with longer peduncles proximally and progressively shorter ones distally. Because Nakai (1944) did not specify the position at which the peduncles were measured, his measurements cannot be directly compared with those of this study, and the reported difference may partly reflect positional variation within the synflorescence.

A critical examination of the five duplicate specimens deposited at TI revealed that the original material represents a mixed collection of two distinct morphological groups. Two sheets (TI00011066 and TI00011067), representing a single individual, are densely covered with hirsute hairs throughout and possess narrowly lanceolate upper bracts. Their flowering state, reaching the terminal head, aligns well with Nakai’s original description and the diagnostic characters of *L. fischeri*.

In contrast, the remaining three specimens (TI00011068, TI00011069, and TI00011070) exhibit glabrous stems and petioles—with only sparse arachnoid hairs on the nodes and bracts—and consistently broadly ovate bracts. These specimens were in the early stages of flowering and showed clear morphological divergence from the aforementioned two sheets. Upon further review, these three sheets were identified as *L. euodon* based on the taxonomic concepts established in this study. Given that the original material of *L. fischeri* var. *spiciformis* comprises more than one taxon, lectotypification is required to fix the application of the name to the element that best reflects the author’s intent.

### Taxonomic treatment

#### Ligularia euodon

Taxon classification

Plantae

AsteralesAsteraceae

Miq., Ann. Mus. Bot. Lugduno-Batavi 3: 188. 1867.

BCC87DC9-C792-5763-A8D3-ED103C69A5A3

≡ Ligularia fischeri var. euodon (Miq.) Kitam., Acta Phytotax. Geobot. 3(3): 170. 1934.
= Ligularia splendens (H.Lév. & Vaniot) Nakai, J. Jap. Bot. 20: 141. 1944. ≡ Senecio splendens H.Lév. & Vaniot, Repert. Spec. Nov. Regni Veg. 8: 139. 1910. Type. Korea, Quelpaert [Jeju-do], in silvis Hallaisan, 600 m et supra, Aug. 1908, *Taquet 995* (Lectotype, first-step designated by Lauener 1976: 371, as ‘holo’; second-step designated here): E! [E00414060]; isolectotypes: E! [E00414059]; LE! [LE01001608]).
= Ligularia kaialpina Kitam., Acta Phytotax. Geobot. 3(3): 170. 1934. Type: Japan. Prov. Kai: mt. Jido, Saino-kawara, 26 Jul. 1931, *S.Kitamura s.n*. (Holotype: TI!).
= Ligularia gigantissima Nakai, J. Jap. Bot. 20: 137. 1944. Type. Japan, Aidzu (會津), 3 Aug. 1879, *Z.Matsumura s.n*. (Holotype: TI! [TI00011071]).
= Ligularia fischeri var. *megalorhiza* Wynn-Jones & J.M.H.Shaw, Hanburyana 5: 52. 2011. Type. Cheju Island [Jeju-do], Korea. Cultivated at Crûg Farm Plants, N. Wales, 31 Aug. 2007, *B.Wynn*-*Jones & S.Wynn*-*Jones 1158* (Holotype: WSY! [WSY0133491]).

##### Type.

Japonia [Japan]. *Siebold s.n*. (Holotype: L! [L0002291]).

##### Diagnosis.

*Ligularia euodon* differs from *L. fischeri* in having stems and petioles glabrous or covered with fine arachnoid hairs (vs. persistently hirsute, with coarse, distinctly septate hairs), leaflike bracts uniformly ovate throughout the synflorescence (vs. ovate proximally and lanceolate distally), and 2–3 supplementary bracts (vs. 2–7).

##### Description.

Perennial herbs, 42.6–156.4 cm tall. Rhizomes short and stout, 2.5–8.1 mm in diameter. Roots fibrous, 15.1–24.2 cm long, 1.0–1.7 mm in diameter. Stems 1.7–9.0 mm in diameter, glabrous or sparsely arachnoid-pubescent. Leaves mostly basal and horizontally spreading; basal leaf blades cordate, 8.1–33.2 × 6.8–36.7 cm, adaxially glabrous, abaxially sparsely hairy, apex obtuse, rounded, or mucronate, base cordate, basal sinus 2.1–8.5 cm deep and 3.8–32.2 cm wide, margin dentate; petioles ridged, 12.8–81.1 cm long, 2.1–7.5 mm wide, glabrous or sparsely arachnoid-pubescent. Cauline leaves gradually reduced upward; petiole bases expanded into sheaths. Synflorescences racemose, 5.2–52.7 cm long, with 4–47 capitula; peduncles in the middle portion of the synflorescence 7.9–18.6 mm long, 0.7–1.4 mm in diameter, sparsely pubescent; leaflike bracts subtending the capitular peduncles uniformly ovate, 12.0–24.9 × 5.6–11.3 mm; supplementary bracts 2–3, lanceolate to oblanceolate, 2.7–16.8 × 1.0–3.1 mm. Involucres cylindrical, 6.7–12.8 mm long, 4.0–4.3 mm in diameter, sparsely pubescent; phyllaries 8–10, free. Receptacles foveolate. Ray florets 4–9; ligules 11.4–28.0 × 2.4–4.9 mm. Disc florets 11–34; corollas 11.4–15.8 mm long, 1.4–3.5 mm in diameter. Achenes 4.6–8.8 mm long, 1.2–1.5 mm in diameter. Pappus 5.8–10.4 mm long, light brown.

##### Korean name.

Ban-deul-ip-gom-chwi (Im 1999).

##### Nomenclatural note.

The taxonomic evidence discussed above indicates that *Ligularia euodon* and *Senecio splendens* are conspecific. Because the name *L. euodon* Miq. (1867) has priority over *S. splendens* H.Lév. & Vaniot, (1910), the basionym of *L. splendens* (H.Lév. & Vaniot) Nakai, *L. euodon* is the correct name for this species.

Léveillé (1910) cited the gathering *Taquet 995* in the protologue of *Senecio splendens* without designating a holotype. Lauener (1976) subsequently referred to the material preserved at E as the “holotype.” Under ICN Art. 9.10, this designation is treated as a first-step lectotypification because the name had not previously been typified. However, the material of *Taquet 995* at E comprises two sheets (E00414059 and E00414060), and Lauener’s citation did not specify which sheet was intended. Therefore, following ICN Art. 9.17, a second-step lectotypification is required. Here, the more complete sheet E00414060 is designated as the lectotype.

#### Ligularia fischeri

Taxon classification

Plantae

AsteralesAsteraceae

(Ledeb.) Turcz., Bull. Soc. Imp. Naturalistes Moscou 11: 95. 1838.

457B8F67-1D0E-512E-A807-F2106CB53B87

≡ Cineraria fischeri Ledeb., Ind. Sem. Hort. Dorpat. 17. 1820. ≡ Senecillis fischeri (Ledeb.) Kitam., Acta Phytotax. Geobot. 8: 82. 1939.
= Ligularia fischeri var. *spiciformis* Nakai, J. Jap. Bot. 20: 138. 1944. Type. Korea, Prov. Kannan (咸南): in herbidis humidis Kantairi (漢岱里), Fusenkôgen, 15 Aug. 1935, *Nakai 15777* (Lectotype, designated here: TI! [TI00011066, TI00011067]).
= Ligularia glabrescens Vorosch., Bull. Princ. Bot. Gard. Acad. Sci. URSS 60: 41. 1965. Type. Russia, Primorsky Krai, in vallis fl. Suifun inter Sofie-Alekseevskoje et Faddeevka (Fadeyevka) in pratis humidis, 16 Jul. 1913, *N.Schipczinsky s.n*. (Holotype: LE! [LE01082339]).
= Ligularia sachalinensis Nakai, J. Jap. Bot. 20: 140. 1944. Type. Russia, Sachalin, Serulako (セルラコ), Aug. 1906, *G.Nakahara s.n*. (Holotype: TI! [TI00011086]), Saghalien Sakaihama, 24 Aug. 1923, *T.Sawada s.n*. (Paratype: TI! [TI00011085]), Saghalin Alexandrovsk [Aleksandrovsk-Sakhalinsky], 19 Aug. 1923, *Okada s.n*. (Paratype: TI! [TI00011084]).

##### Type.

Russia, culta, 20. Jul. year unknown, *Hb. Meyer s.n*. (Neotype, designated by Illarionova 2013, p. 1154: LE! [LE01010001, LE01010002]).

##### Diagnosis.

*Ligularia fischeri* differs from *L. euodon* in its persistently hirsute stems and petioles, with coarse, distinctly septate hairs often multiseriate at the base (vs. glabrous or finely arachnoid), leaflike bracts ovate in the proximal portion of the synflorescence and lanceolate in the distal portion (vs. uniformly ovate), and 2–7 supplementary bracts (vs. 2–3).

##### Description.

Perennial herbs, 92.4–154.3 cm tall. Rhizomes short and stout, 8.2–13.5 mm in diameter. Roots fibrous, 1.2–11.2 cm long, 0.9–1.3 mm in diameter. Stems 3.2–7.4 mm in diameter, densely and persistently hirsute. Leaves mostly basal and horizontally spreading; basal leaf blades cordate, 8.1–19.8 × 8.6–21.6 cm, adaxially glabrous, abaxially densely hairy, apex obtuse, rounded, or mucronate, base cordate, basal sinus 2.3–6.0 cm deep and 6.5–17.2 cm wide, margin dentate; petioles ridged, 11.5–45.2 cm long, 1.5–4.9 mm wide, densely hirsute. Cauline leaves gradually reduced upward; petiole bases expanded into sheaths. Synflorescences racemose, 9.8–29.3 cm long, with 10–46 capitula; peduncles in the middle portion of the synflorescence 10.3–47.9 mm long, 0.7–1.2 mm in diameter, densely hirsute; bracts ovate proximally, becoming lanceolate distally, 19.2–35.3 × 7.0–14.2 mm; supplementary bracts 2–7, lanceolate, oblanceolate, or linear, 8.1–17.0 × 0.9–2.7 mm. Involucres cylindrical, 9.7–11.7 mm long, 4.2–6.2 mm in diameter, densely pubescent; phyllaries 8–10, free. Receptacles not observed. Ray florets 8–10; ligules 15.1–24.3 × 3.4–5.2 mm. Disc florets 23–34; corollas 9.9–14.2 mm long, 1.1–2.2 mm in diameter. All achenes observed in the examined specimens were immature or apparently abortive; therefore, mature achene dimensions could not be determined. Pappus 10.2–11.0 mm long, light brown.

##### Korean name.

Gom-chwi (Chung 1957; Lee 1979; Lee 1996a, 1996b; Im 1999), Wang-gom-chwi (Park 1974).

##### Nomenclatural note.

The gathering *Nakai 15777* is mixed. The lectotype designated here is represented by two sheets (TI00011066 and TI00011067) that belong to a single individual consistent with the protologue and the diagnostic characters of *L. fischeri* var. *spiciformis*. In contrast, the remaining three sheets (TI00011068, TI00011069, and TI00011070) represent different individuals identified here as *L. euodon*. Accordingly, these three sheets are not considered part of the type material of *L. fischeri* var. *spiciformis*.

## Conclusion

Based on type specimens and comprehensive micromorphological and nomenclatural evidence, the taxonomic complexities of the *Ligularia fischeri* complex in Korea are resolved, refined species circumscriptions are established, and critical lectotypifications (including second-step lectotypifications) are provided alongside updated synonymies under the ICN.

## Supplementary Material

XML Treatment for Ligularia euodon

XML Treatment for Ligularia fischeri

## Additional information

### Artificial Intelligence (AI) use

The authors accept full responsibility for the content of the manuscript, including the disclosure of any use of AI.

Regarding the use of AI in the preparation of this manuscript, the authors declare the following: Gemini

Used for: Language, style and writing

### Author ORCIDs

M. Park https://orcid.org/0000-0002-9070-6669

N. Na https://orcid.org/0000-0003-1437-2393

C.-g. Jang https://orcid.org/0000-0002-3013-9068

### Data availability

All of the data that support the findings of this study are available in the main text or Supplementary Information.

## Supplementary materials

Supplementary material 1 Images of type materials for the Ligularia fischeri complex (Asteraceae) and related taxa Data type pdf Explanation note This supplementary PDF file contains high-resolution images of key type specimens examined for the nomenclatural and taxonomic study of the *Ligularia fischeri* complex (Asteraceae). It includes holotypes, lectotypes (including first- and second-step lectotypifications), and isolectotypes deposited in major herbaria (e.g., E, L, LE, TI). These images provide critical morphological and nomenclatural evidence supporting the updated synonymies and typifications presented in the manuscript. https://binary.pensoft.net/file/1826858This dataset is made available under the Open Database License (http://opendatacommons.org/licenses/odbl/1.0/). The Open Database License (ODbL) is a license agreement intended to allow users to freely share, modify, and use this Dataset while maintaining this same freedom for others, provided that the original source and author(s) are credited. Minsu Park, Nuree Na, Chang-gee Jang

Supplementary material 2 Specimens examined in the taxonomic revision of the Ligularia fischeri complex Data type xlsx Explanation note This dataset provides a list of herbarium specimens examined in the taxonomic revision of the *Ligularia fischeri* complex. It includes scientific names, collection localities, collection dates, collector information, and herbarium acronyms. https://binary.pensoft.net/file/1826859This dataset is made available under the Open Database License (http://opendatacommons.org/licenses/odbl/1.0/). The Open Database License (ODbL) is a license agreement intended to allow users to freely share, modify, and use this Dataset while maintaining this same freedom for others, provided that the original source and author(s) are credited. Minsu Park, Nuree Na, Chang-gee Jang
