# Supplementary material for: Taxonomic revision of the Ligularia fischeri complex (Asteraceae) in Korea
Source: PhytoKeys. 2026 Nov 2;281:65–76. doi: 10.3897/phytokeys.281.211143 (PMC13749061; doi:10.3897/phytokeys.281.211143)
Supplement: Supplementary file 1 [file phytokeys-281-065_article-211143__-s001.pdf]

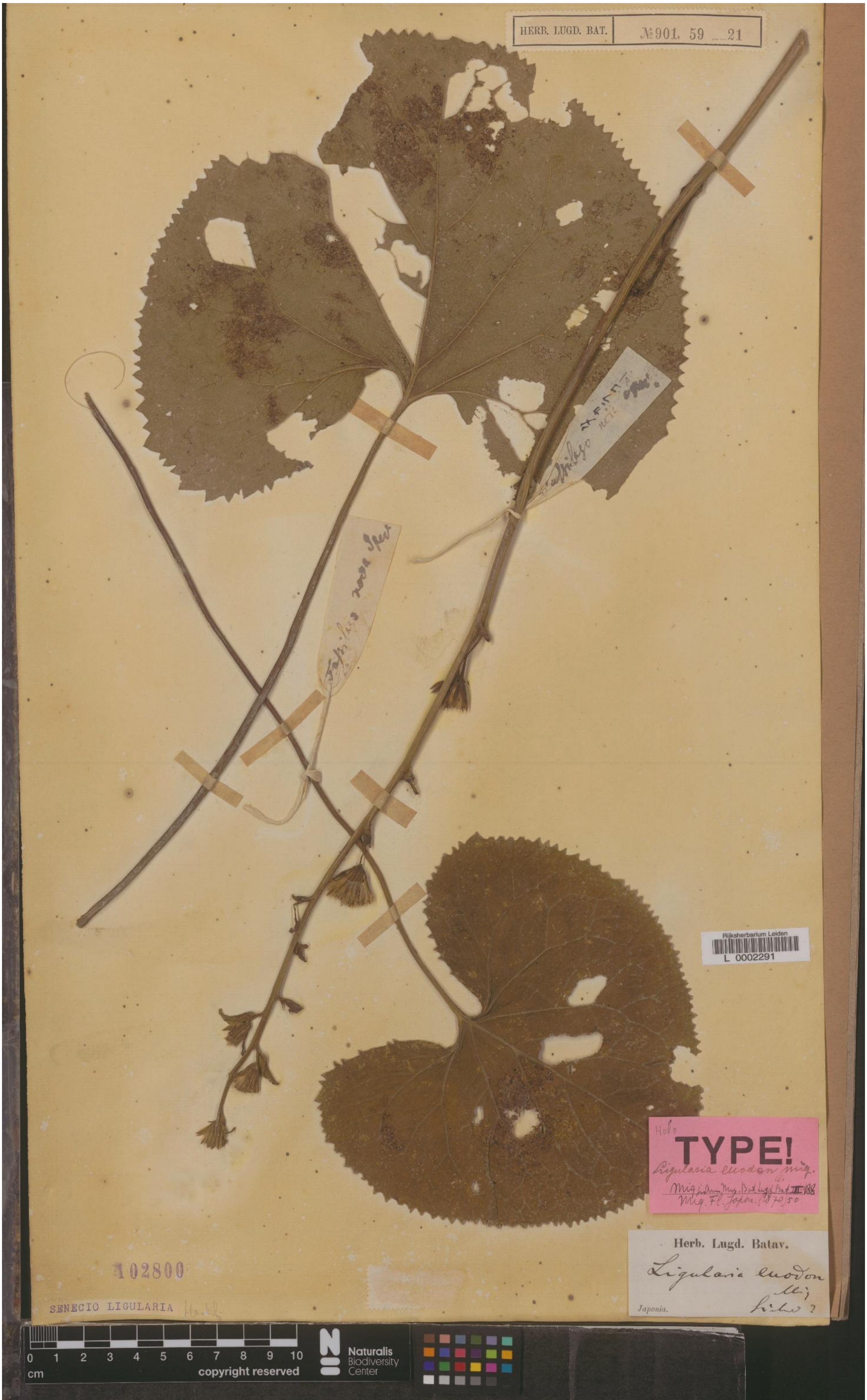

Type material of *Ligularia euodon* Miq. Japonia [Japan]. *Siebold s.n.* (Holotype: L [L0002291])

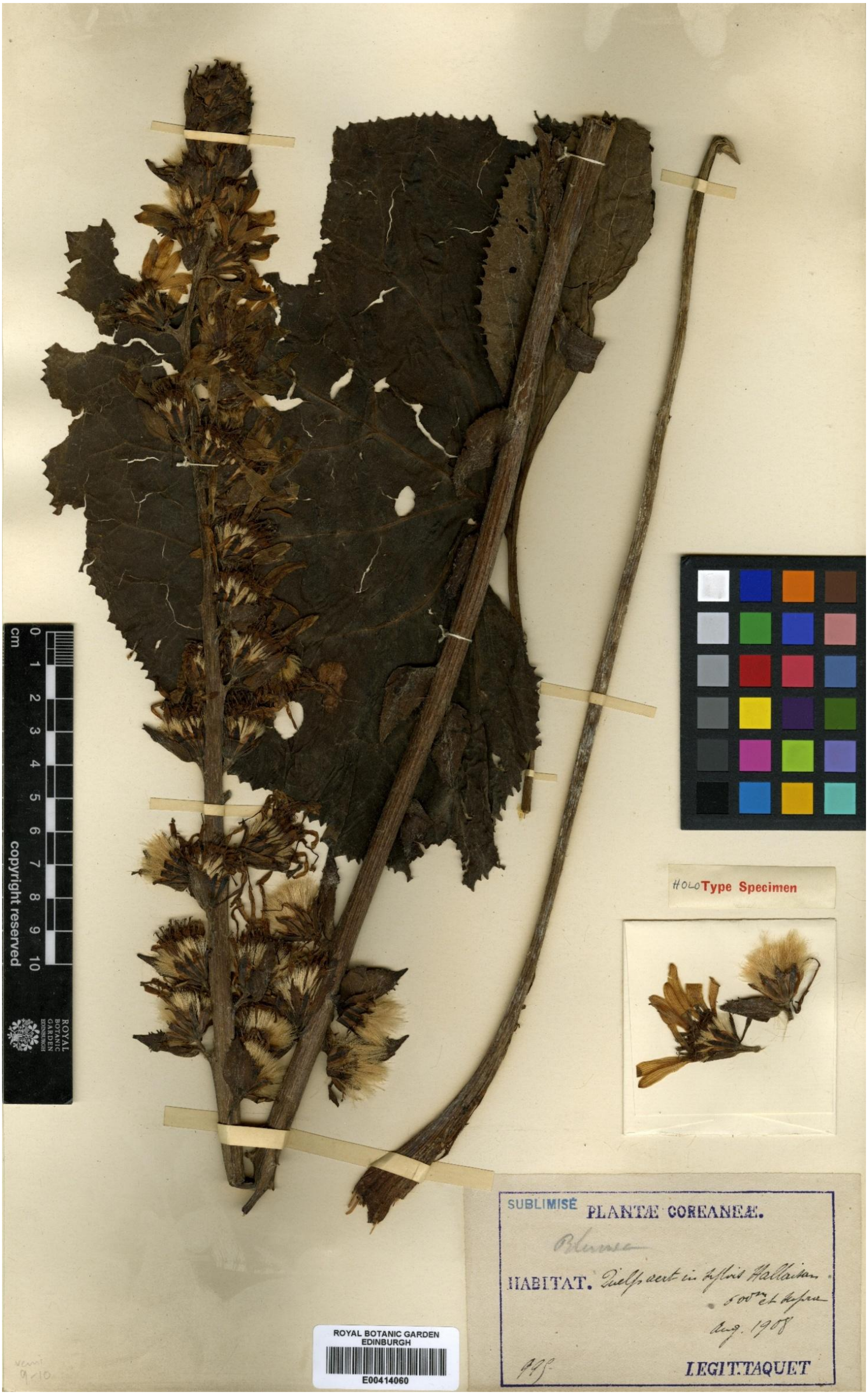

Type material of *Senecio splendens* H.Lév. & Vaniot. Korea, Quelpaert [Jeju–do], in silvis Hallaisan, 600 m et supra, Aug. 1908, *Taquet 995* (Lectotype: E [E00414060]).

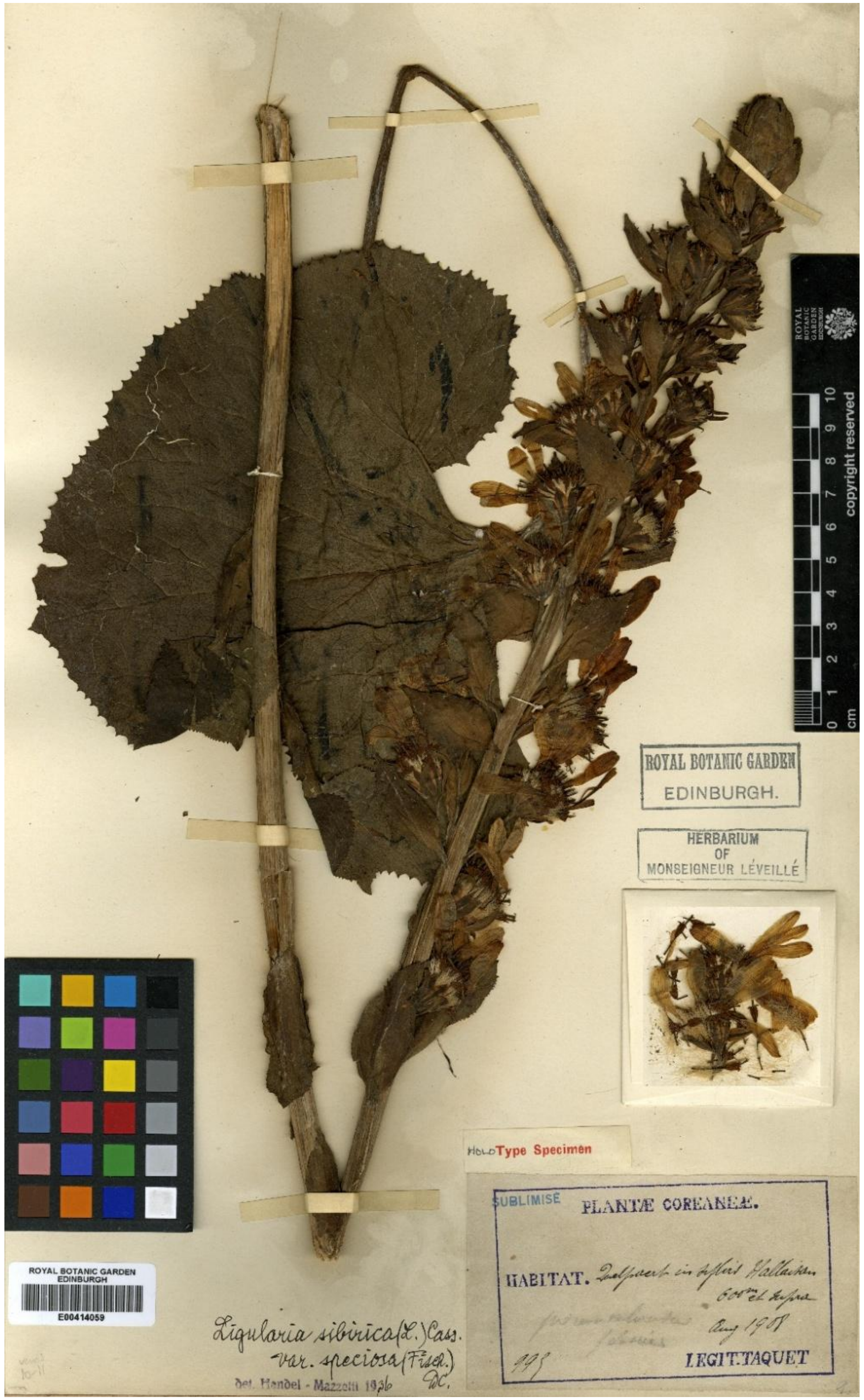

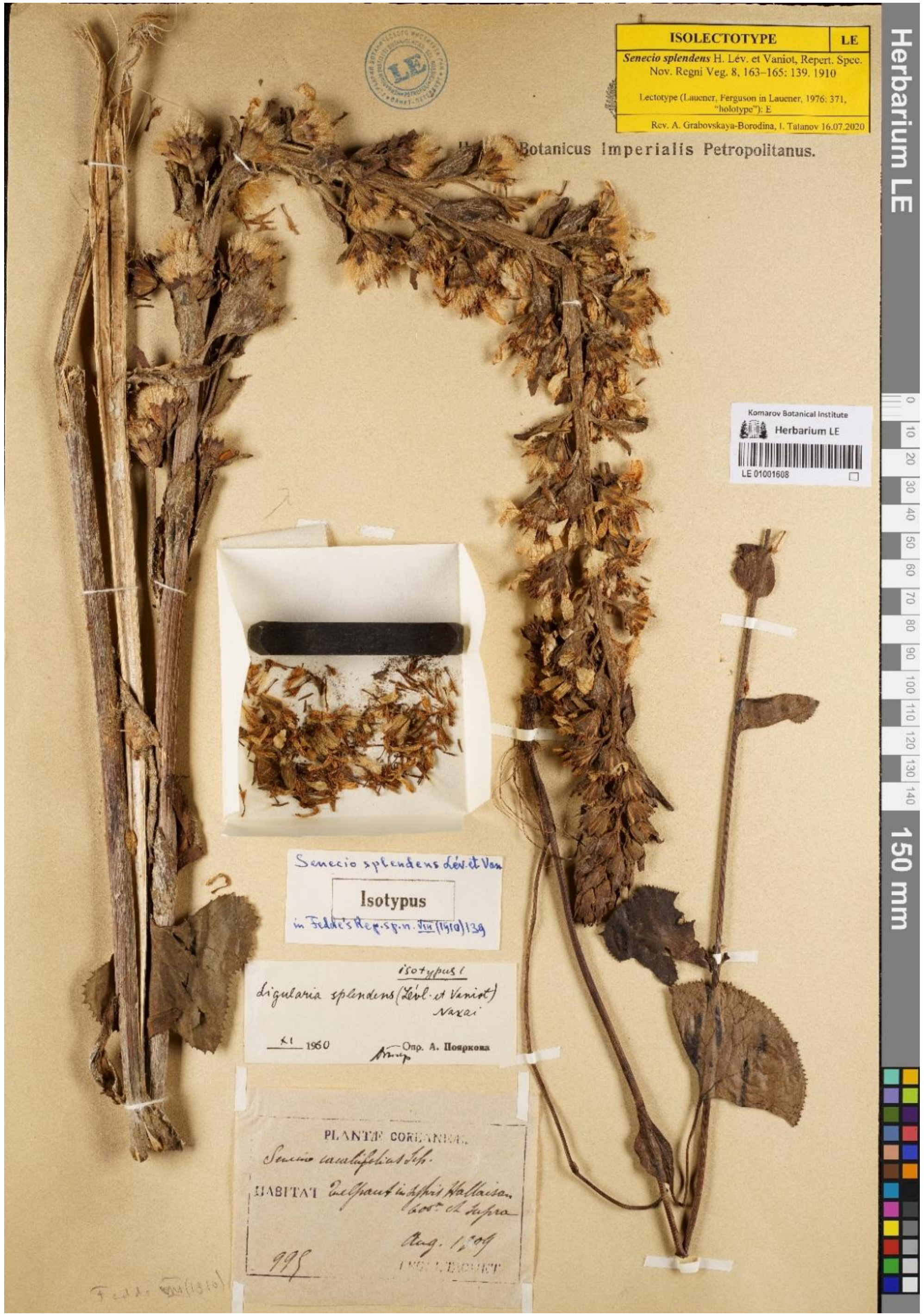

Type material of *Senecio splendens* H.Lév. & Vaniot. Korea, Quelpaert [Jeju–do], in silvis Hallaisan, 600 m et supra, Aug. 1908, *Taquet 995* (Isolectotype: E [E00414059]; LE [LE01001608]).

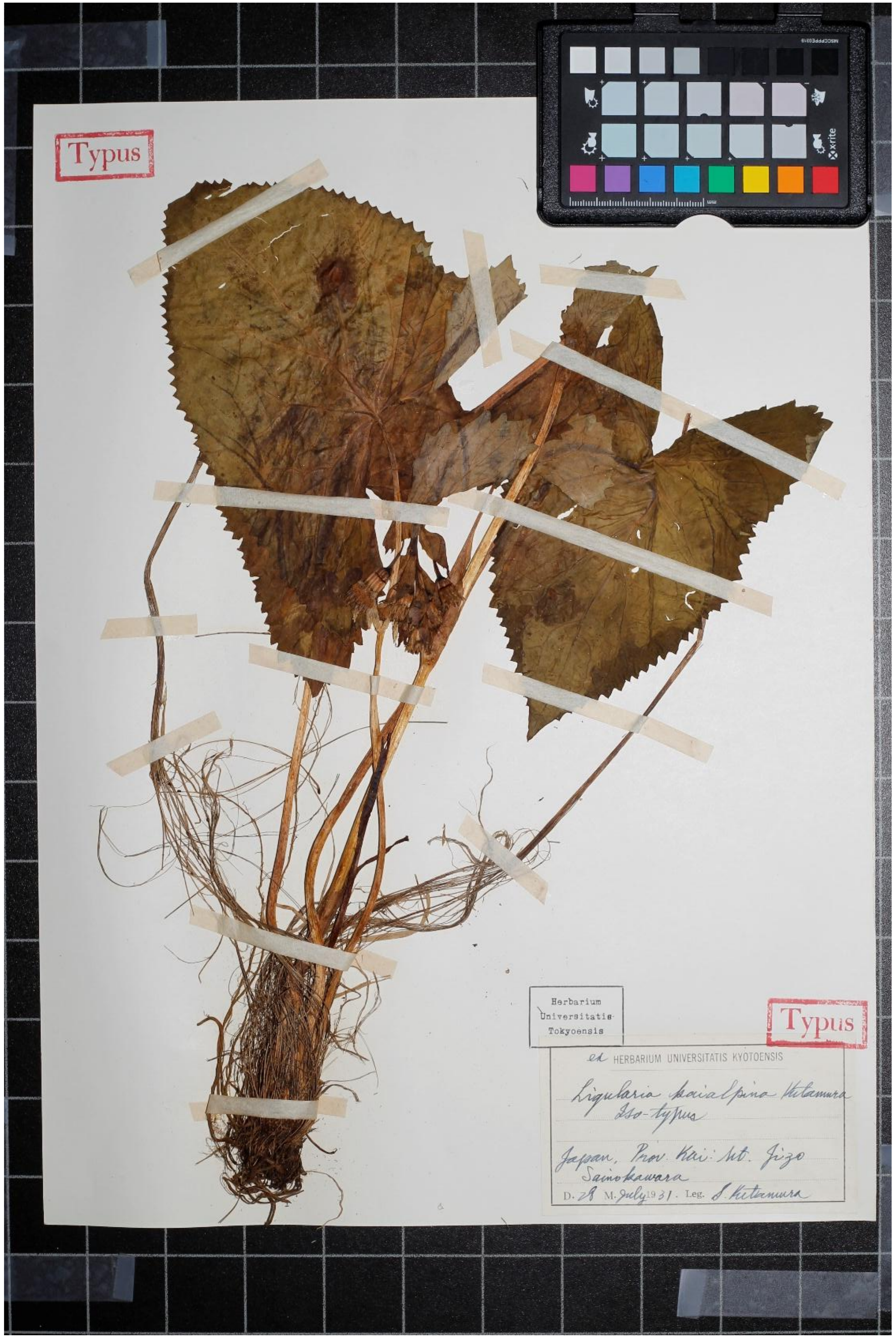

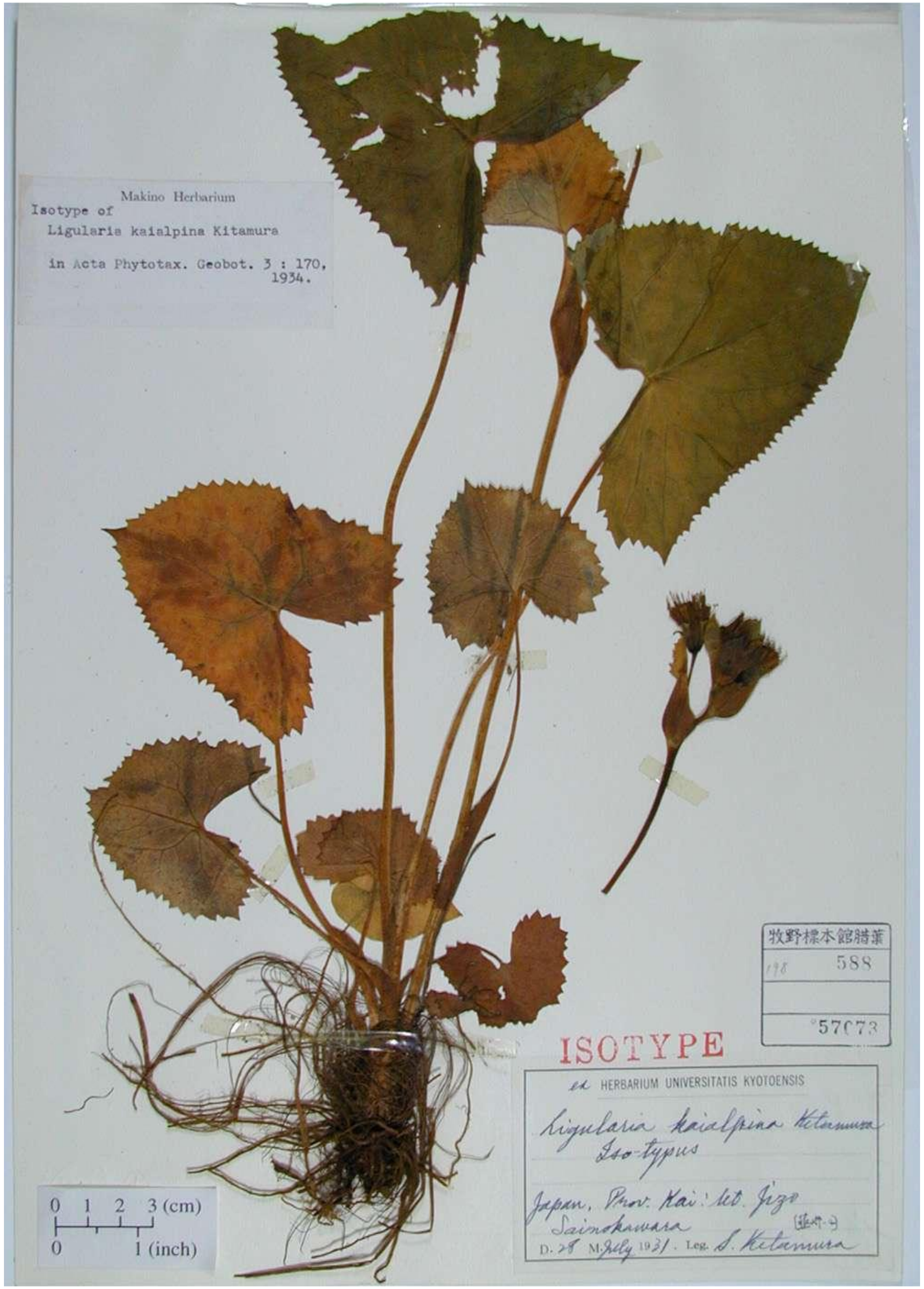

Type material of *Ligularia kaialpina* Kitam. Japan. Prov. Kai: mt. Jido, Saino–kawara, 26 Jul. 1931, *S.Kitamura s.n.* (Holotype: TI [?], Isotype: MAK [?])

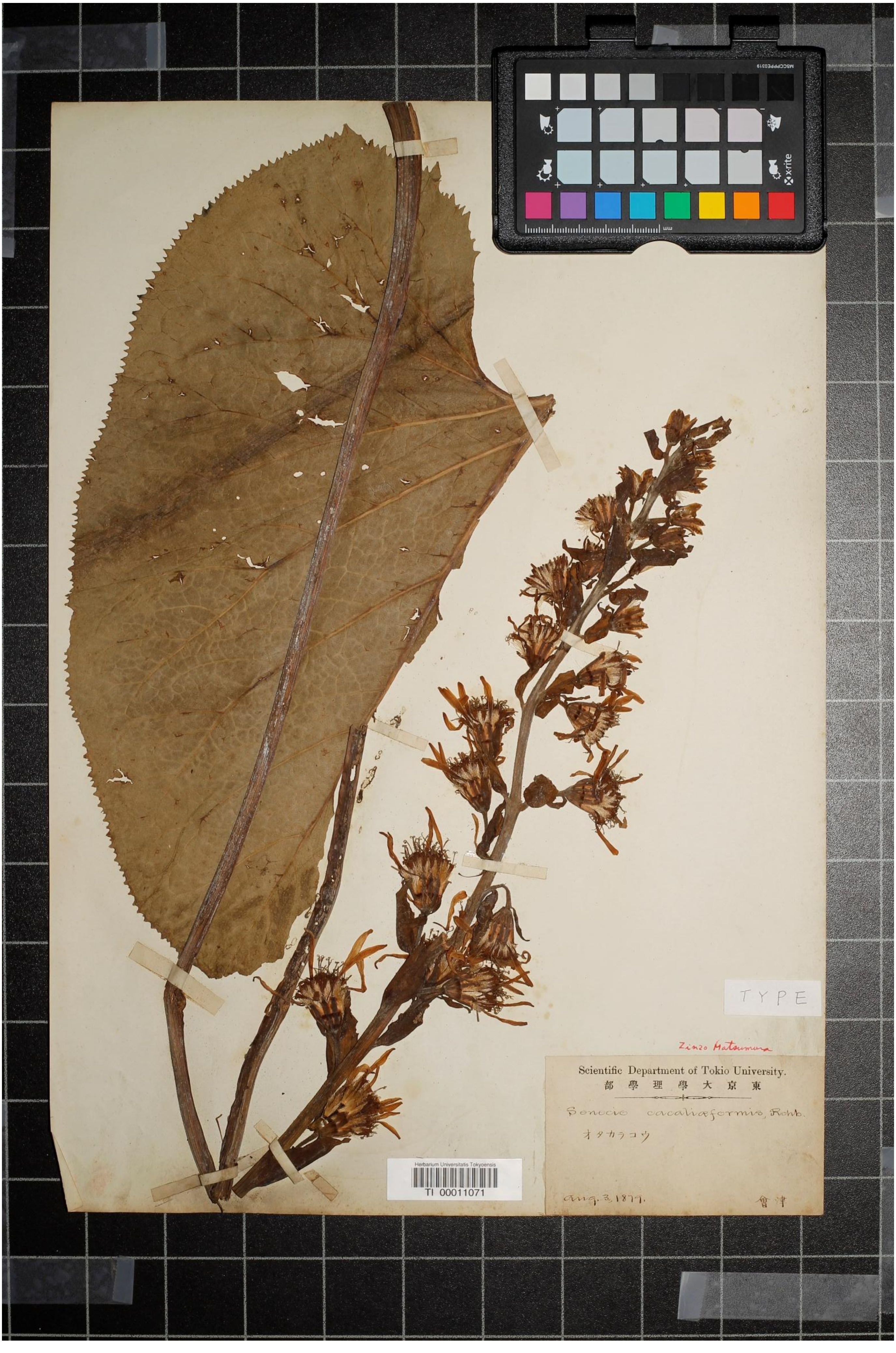

Type material of *Ligularia gigantissima* Nakai. Japan, Aidzu (會津), 3 Aug. 1879, *Z.Matsumura s.n.* (Holotype: TI [TI00011071])

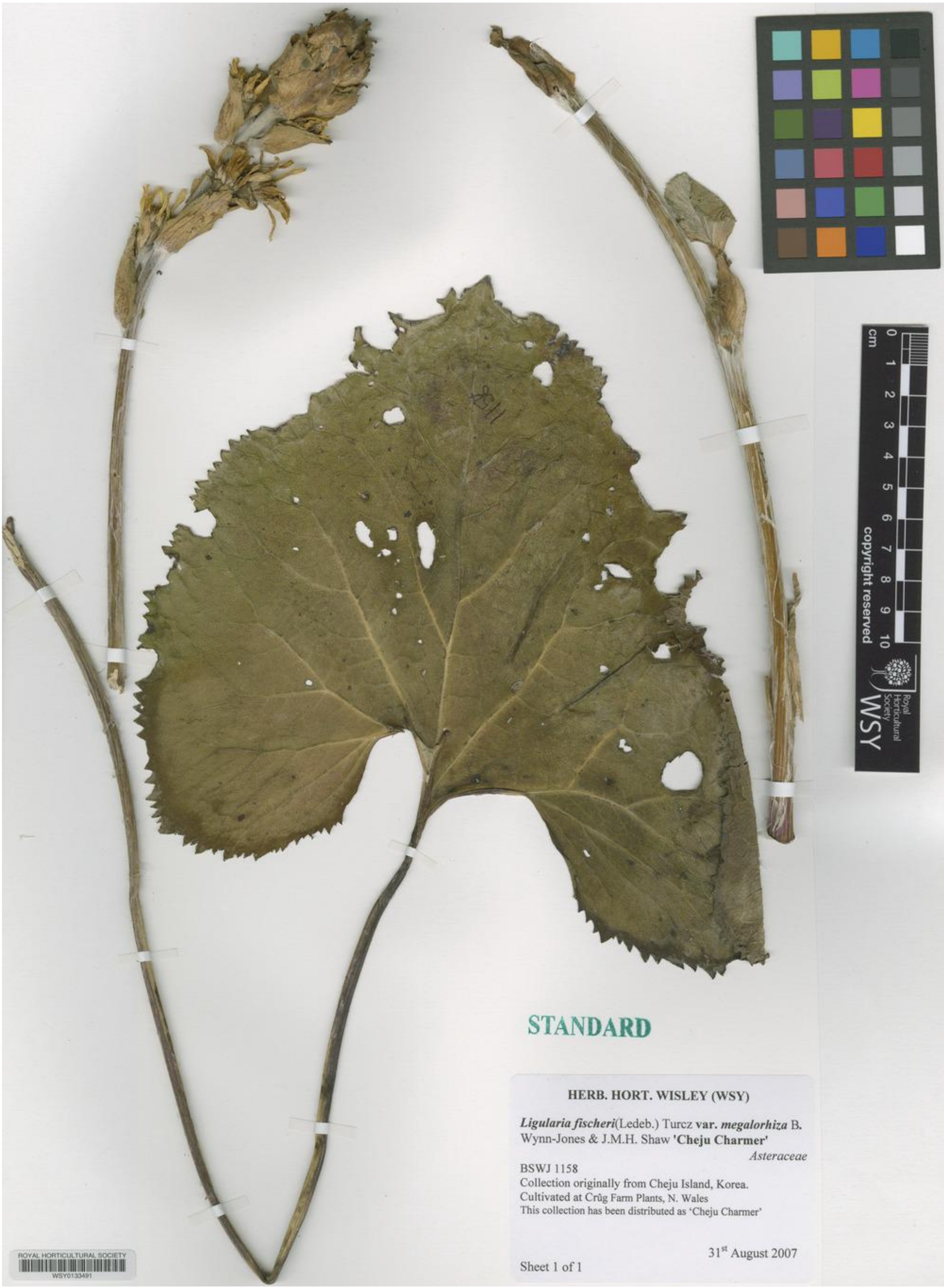

Type material of *Ligularia fischeri* var. *megalorhiza* Wynn–Jones & J.M.H.Shaw. Cheju Island [Jeju–do], Korea. Cultivated at Crûg Farm Plants, N. Wales, 31 Aug. 2007, *B.Wynn–Jones & S.Wynn–Jones 1158* (Holotype: WSY [WSY0133491])

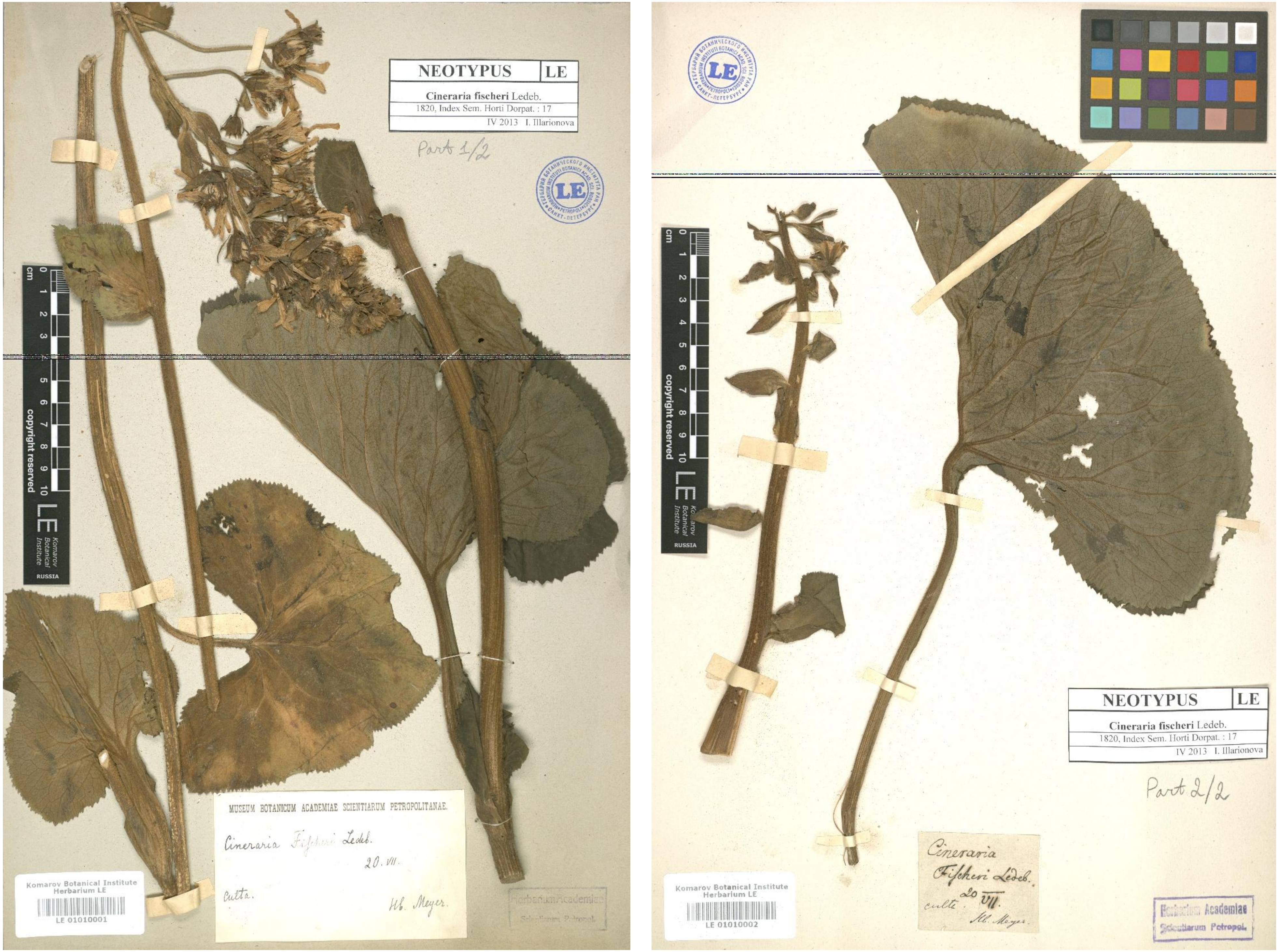

Type material of *Cineraria fischeri* Ledeb. Russia, culta, 20. Jul. year unknown, *Hb. Mayer s.n.* (Neotype: LE [LE01010001, LE01010002])

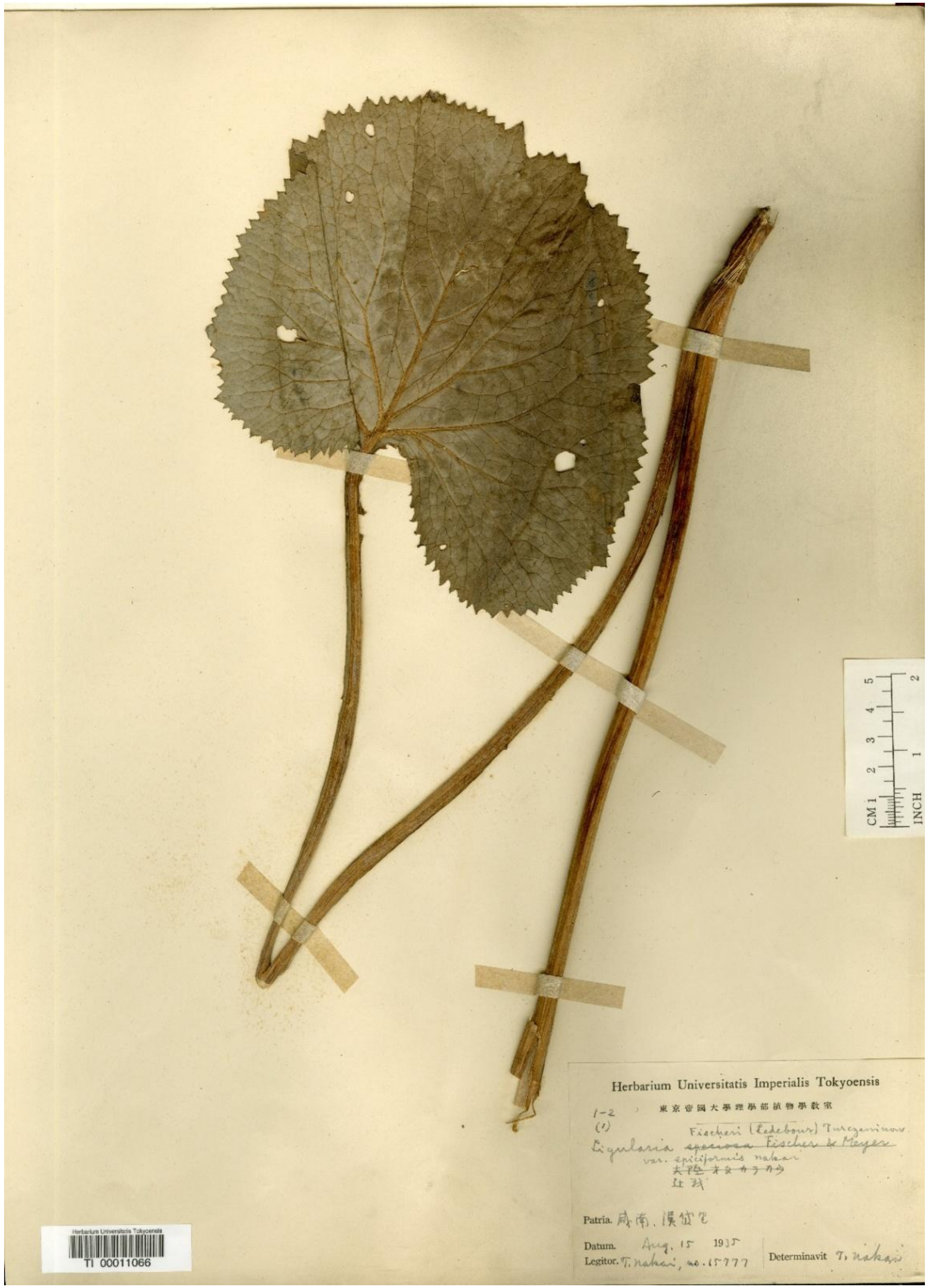

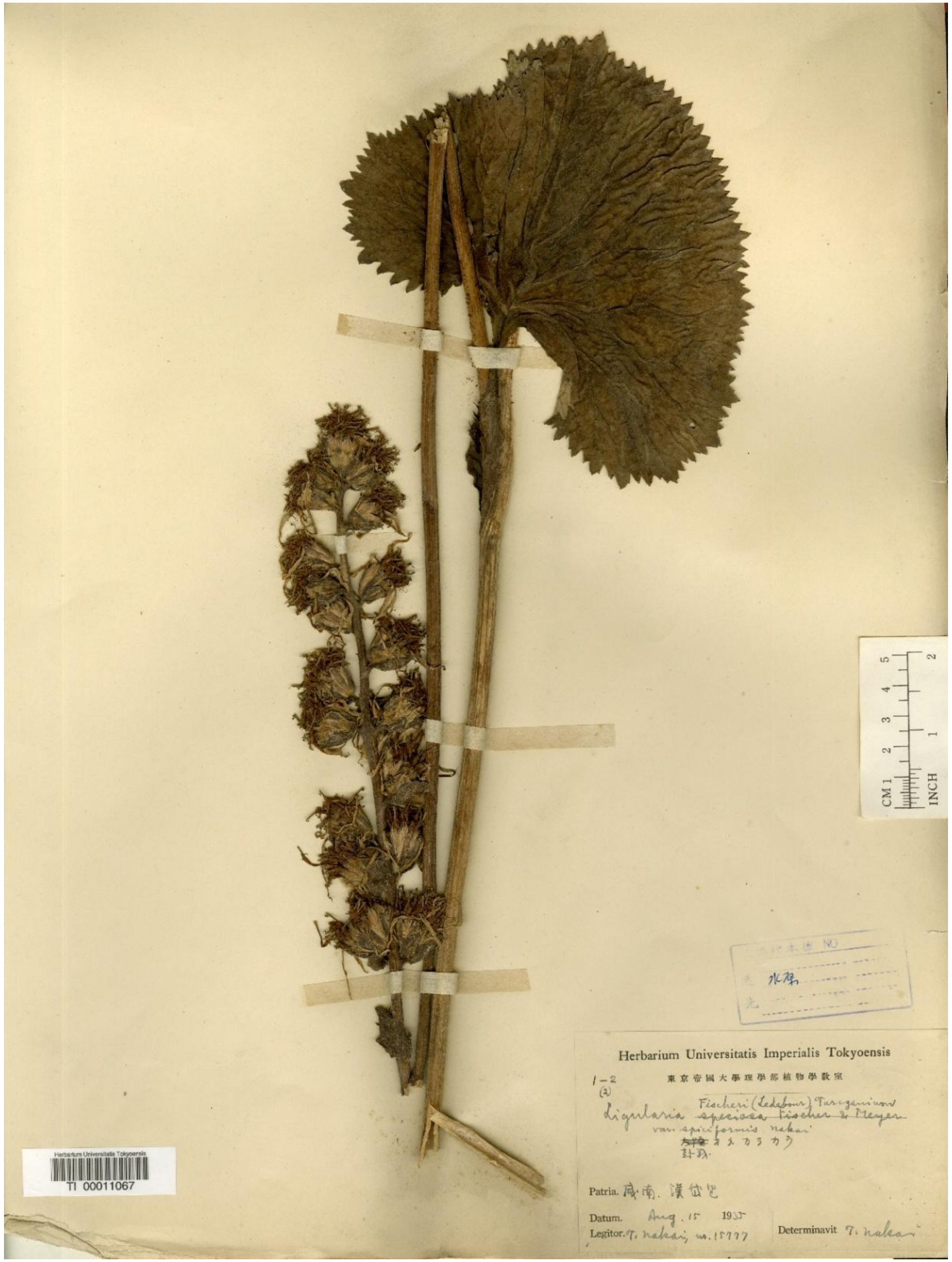

Type material of *Ligularia fischeri* var. *spiciformis* Nakai. Korea, Prov. Kannan (咸南): in herbidis humidis Kantairi (漢岱里), Fusenkôgen, 15 Aug. 1935, *Nakai 15777* (Lectotype: TI [TI00011066, TI00011067])

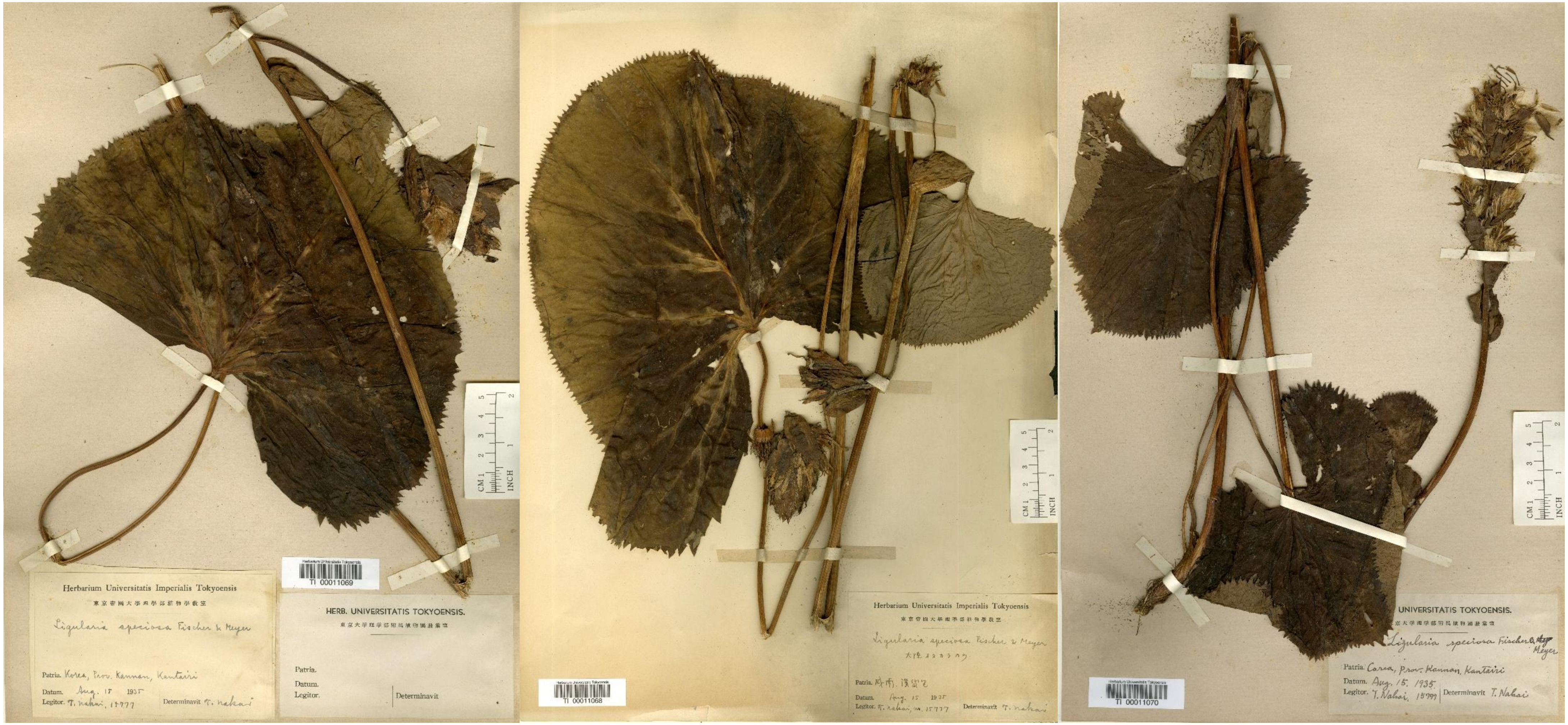

Type material of *Ligularia fischeri* var. *spiciformis* Nakai. Korea, Prov. Kannan (咸南): in herbidis humidis Kantairi (漢岱里), Fusenkôgen, 15 Aug. 1935, *Nakai 15777* (reidentified as *L. euodon* Miq. in this study: TI [TI00011068, TI00011069, TI00011070])

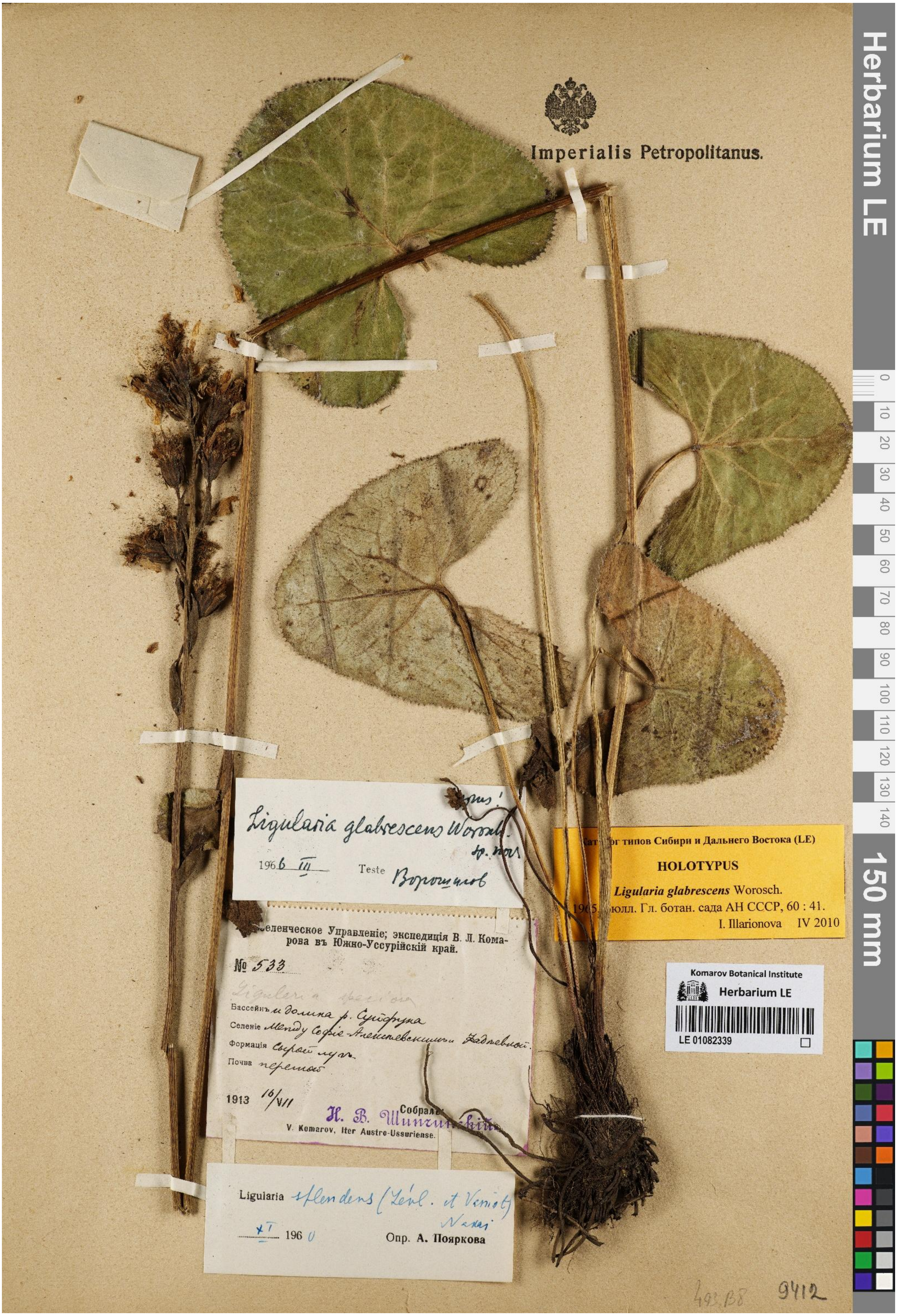

Type material of *Ligularia glabrescens* Vorosch. Russia, Primorsky in vallis fl. Suifun inter Sofie–Alekseevskoje et Faddeevka (Fadeyevka) in pratis humidis. 16 Jul. 1913, *N.Schipczinsky s.n.* (Holotype: LE [LE01082339])

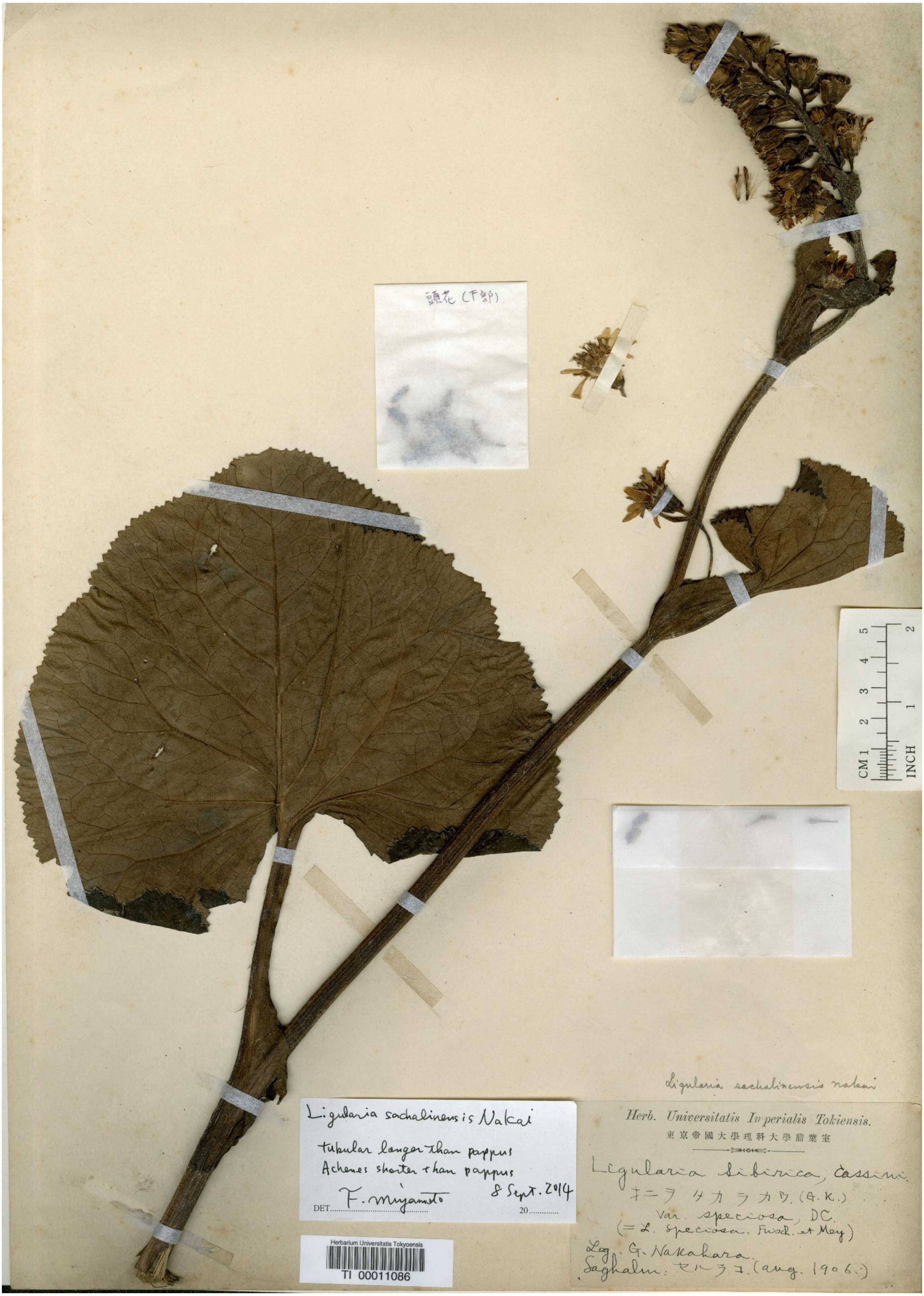

Type material of *Ligularia sachalinensis* Nakai. Russia, Sachalin, Serulako (セルラコ), Aug. 1906, *G.Nakahara s.n.* (Holotype: TI [TI00011086])

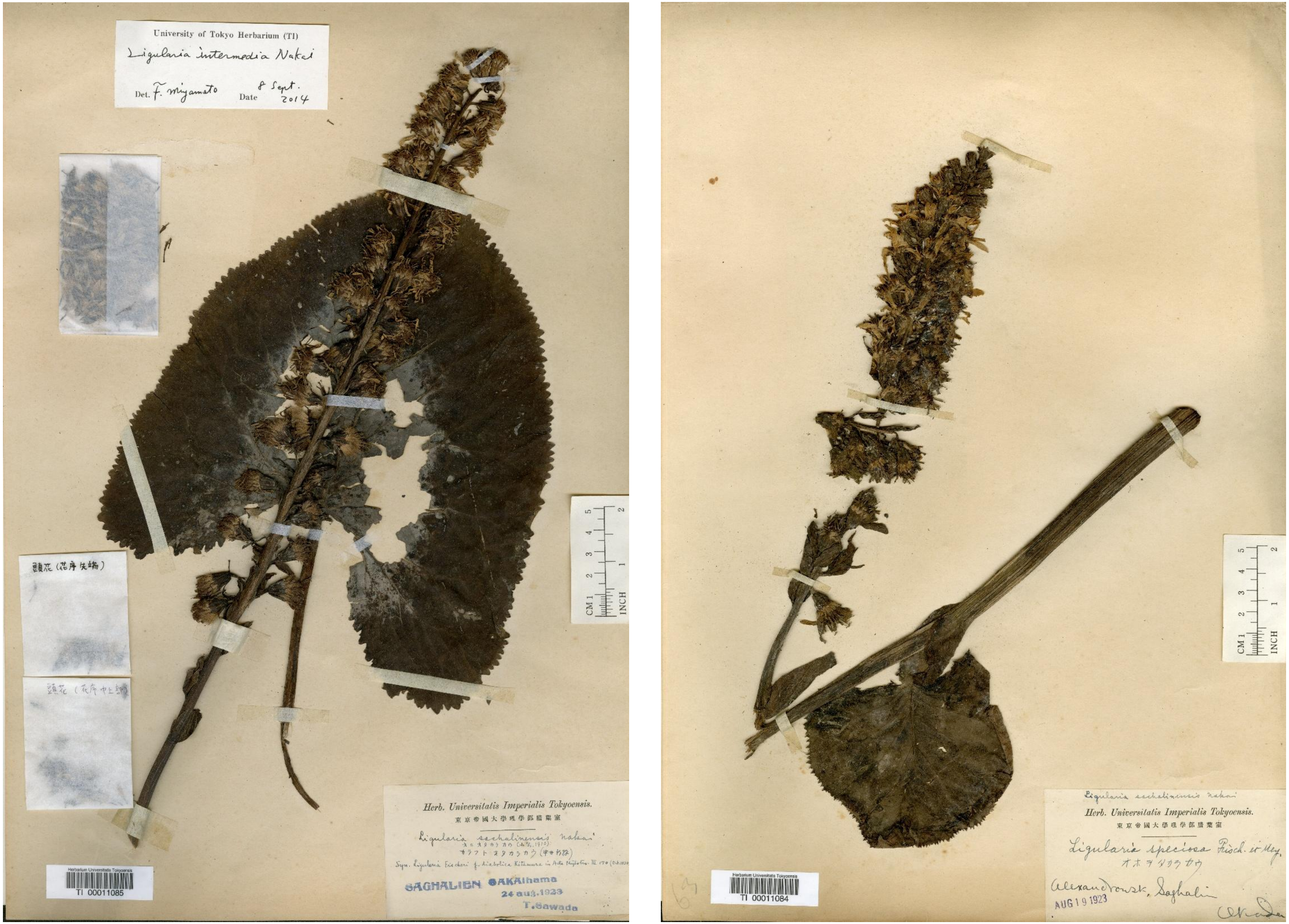

Type material of *Ligularia sachalinensis* Nakai. Russia, Saghalien Sakaihama, 24 Aug. 1923, *T.Sawada s.n.* (Paratype: TI [TI00011085]); Saghalin Alexandrovsk, 19 Aug. 1923, *Okada s.n.* (Paratype: TI [TI00011084])
